# Flight initiation by Ferruginous Hawks depends on disturbance type, experience, and the anthropogenic landscape

**DOI:** 10.1371/journal.pone.0177584

**Published:** 2017-05-18

**Authors:** Cameron J. Nordell, Troy I. Wellicome, Erin M. Bayne

**Affiliations:** 1 Department of Biological Sciences, University of Alberta, Edmonton, Canada; 2 Environment Canada, Edmonton, Alberta, Canada; Liverpool John Moores University, UNITED KINGDOM

## Abstract

The expansion of humans and their related infrastructure is increasing the likelihood that wildlife will interact with humans. When disturbed by humans, animals often change their behaviour, which can result in time and energetic costs to that animal. An animal's decision to change behaviour is likely related to the type of disturbance, the individual's past experience with disturbance, and the landscape in which the disturbance occurs. In southern Alberta and Saskatchewan, we quantified probability of flight initiation from the nest by Ferruginous Hawks (*Buteo regalis*) during approaches to nests by investigators. We tested if probability of flight was related to different disturbance types, previous experience, and the anthropogenic landscape in which individual Ferruginous Hawks nested. Probability of flight was related to the type of approach by the investigator, the number of previous visits by investigators, and the vehicular traffic around the nest. Approaches by humans on foot resulted in a greater probability of flight than those in a vehicle. Approaches in a vehicle via low traffic volume access roads were related to increased probability of flight relative to other road types. The number of previous investigator approaches to the nest increased the probability of flight. Overall, we found support that Ferruginous Hawks show habituation to vehicles and the positive reinforcement hypotheses as probability of flight was negatively related to an index of traffic activity near the nest. Our work emphasizes that complex, dynamic processes drive the decision to initiate flight from the nest, and contributes to the growing body of work explaining how responses to humans vary within species.

## Introduction

Anthropogenic habitat alteration is a primary driver of wildlife population declines [1]. In landscapes where habitat has been altered by humans, there is often an increase in the frequency of human-wildlife interactions [2, 3]. In theory, animal responses to humans should resemble those to potential predators [4]. There is a conservation concern when these interactions result in increases in energetic costs for wildlife. For example, individuals may increase defence, aggression, or vigilance behaviours, thus decreasing time and energy available for foraging, offspring care, maintenance, and rest [5–8]. Understanding when and how wildlife responds to human disturbances, and whether unnecessary behavioural changes can be mitigated are important components of human-wildlife coexistence [9–11]

The decision to initiate flight in response to a disturbance stimulus is determined by a cost / benefit trade-off whereby the perceived risk (cost) associated with an approaching threat becomes greater than the benefit of maintaining regular activity [8, 12]. Animals continually assess risk when interacting with humans or predators, ultimately responding behaviourally when it is optimal to do so based on their perception of risk to themselves and their young [13]. Flight initiation distance (FID) provides an unambiguous and easily quantifiable change in animal behaviour [14], and presumably differs because of differences in the assessment of costs and benefits among individuals. However, [15] stressed that responses to disturbance stimuli are driven not only by inter-individual biological differences, but by the properties of the disturbance itself and the environment in which the interaction occurs.

FID is known to change with the presence of young [16], distance to refuge [17], group size [8], concealment, visibility [17, 18], and weather [19]. However, controlling for these factors, FID still varies considerably within a species [9]. An increasing number of studies demonstrate that differences between approaching stimuli can change FID. It has been suggested that faster moving, noisier, and larger stimuli [11, 20, 21], are perceived as greater risk. This dangerous stimulus hypothesis predicts that vehicles should result in greater FID than humans on foot. However, FIDs in response to vehicles have been shown to be shorter for some species than those in response to humans on foot [22–25], perhaps owing to habituation to vehicles which are more commonly observed than humans on foot.

In addition to the nature of the stimuli, an animal's past experiences with stimuli [26], are potentially important components of FID. However, few studies have quantified FID using repeated stimulation (but see [27]). For many species it remains unclear how FIDs change in response to repeated human disturbance. FID tends to decrease as the level of anthropogenic disturbance in a region increases [28–35], potentially owing to greater habituation by animals living in areas with more disturbance. [36] quantified the number of pedestrians at a given site and found a negative relationship with FIDs in urban birds. However, given the difficulty of quantifying human-wildlife interactions at large scales, quantitative links between the number of human-wildlife interactions in anthropogenic landscapes and FID are deficient [33]. No studies have examined the effects of disturbance type and animal experience in different landscapes simultaneously.

We created three models to determine if flight initiations in Ferruginous Hawks (*Buteo regalis*) were most strongly influenced by: 1) the type of disturbance stimulus (vehicle approach on different road types relative to humans on foot); 2) the individual's previous experience with disturbance; and 3) the amount of human footprint in the landscape they nested in. Nesting in isolated trees in open grassland habitat, most nests are relatively poorly concealed and adults likely detect humans and initiate flight at great distances, presumably to deter potential threats from approaching the nest and young [37, 38]. Ferruginous Hawks breed across southern Alberta and Saskatchewan and are reportedly sensitive to human disturbance [39, 40]. Furthermore, the species was designated as nationally Threatened in Canada under the Species At Risk Act [41] in 2009 [42]. The perception that Ferruginous Hawks are sensitive to human disturbance and their listed status, has resulted in the use of large setback distances as a primary conservation strategy for the species in Canada [43]. Thus, understanding how different human activities influence FID is directly relevant to current conservation strategies for this species.

## Methods

### Ethics statement

Our study involved observation of a protected species. All reasonable measures were taken to limit harm to individuals, as outlined in the methods below. Our study was compliant with the Ethical Treatment of Animals Guidelines under University of Alberta Animal Care #724, Permit AUP00000018. Land access permissions were acquired from appropriate governing bodies, including on federal (e.g. Canadian Forces Base Suffield, Prairie Farm Rehabilitation Administration), provincial (e.g. community pastures) and, private land (e.g. private citizen and corporate landowners), before approaching nests.

### Study area

Data collection for this study was conducted across the mixed and moist-mixed grasslands ecoregion from southwestern Alberta to southeastern Saskatchewan. This area spans ~900 km east to west and ~300 km north south, with a total area > 250 000 km^2^. Ferruginous Hawk habitat is typified by grassland with minimal topography and few hills between 600 and 1300 m above sea level. In this region, Ferruginous Hawks generally nests in lone trees (but occasionally in tree stands), free standing artificial nesting platforms [44], and occasionally electrical transmission infrastructure towers. Dominant nest tree species are aspen and cottonwood (*Populus* spp.). The region has undergone considerable landscape transformation since it was first settled in the 1900s [45–47]. Ferruginous Hawks nest exclusively in non-urban regions [48], but do so across a gradient of human activity. Active oil and gas well densities range from 0–15 wells per km^2^. Land-cover conversion around nest sites has resulted in local vegetation that ranges from 0 to 100% agriculture, a mix of cropland and rangeland pasture [46]. A network of roads with varying traffic volumes permeates the study region, including variable densities of highways, range roads along most township gridlines (1.6 km separation), and industrial access roads, right of way, and private access roads (henceforth access roads) which allow vehicular access near many nests. Highways and some range roads are paved, most range roads are gravel, and access roads are gravel, graded dirt, or vehicle tracks worn into the ground. Anthropogenic features in Alberta (e.g. industrial infrastructure, crop fields, and houses) are accompanied by increases in roads and traffic [49], so we used traffic as a surrogate index for total level of anthropogenic activity around nests.

### Data collection

**Fig 1:**
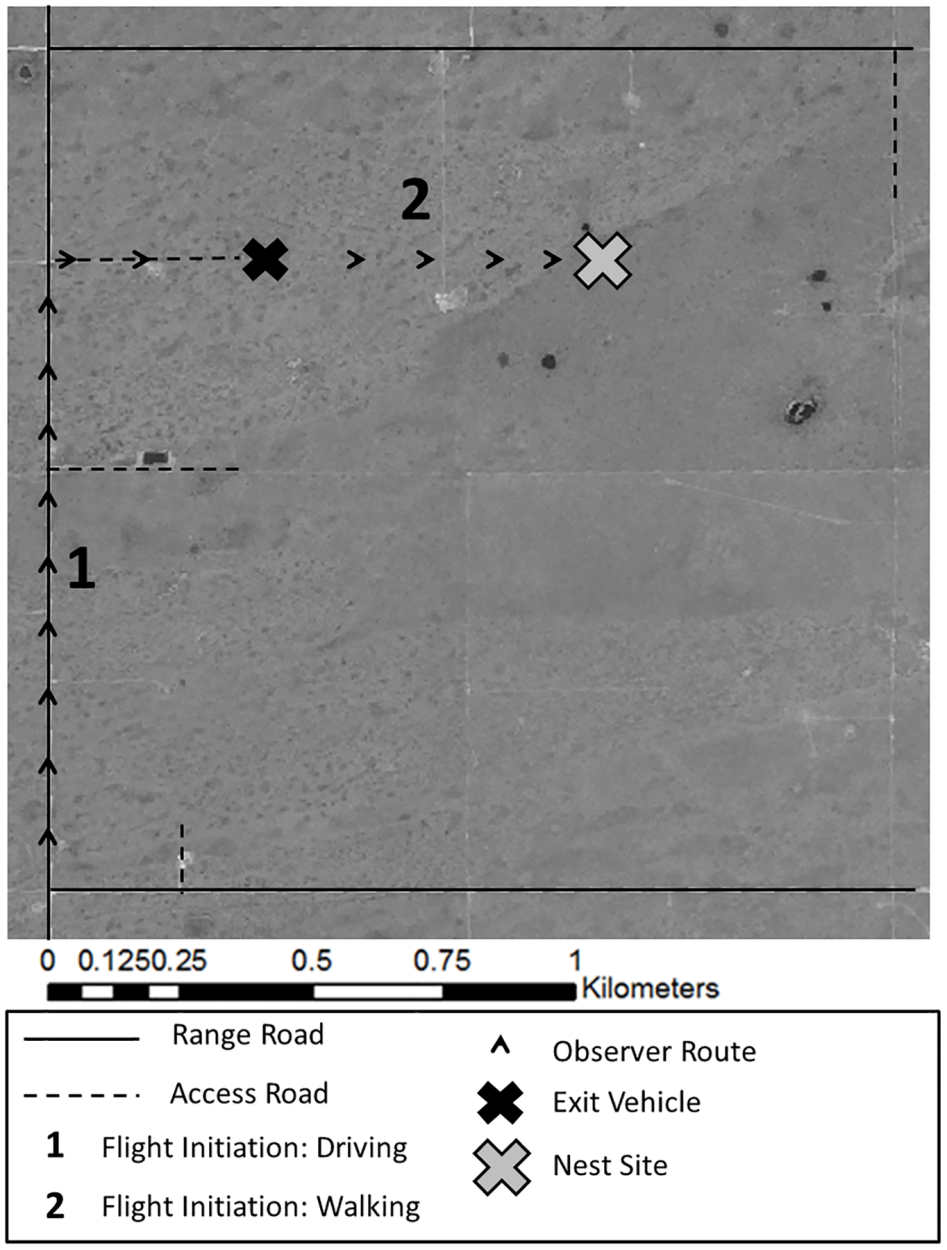
A visual representation of a typical investigator approach to a Ferruginous Hawk nest in southern Alberta and Saskatchewan during the breeding season in 2012, 2013 or 2014. Investigators travelled in a vehicle via publicly available roads before they exited the vehicle and, when granted permission to access the land, proceeded the remainder of the distance to the nest on foot. (1) and (2) indicate two hypothetical approaches in which Ferruginous Hawks initiated flight from the nest while the investigator was driving or walking, respectively.

We documented FID beginning in 2012 as part of a large scale Ferruginous Hawk nest monitoring project across the Canadian mixed and moist-mixed grasslands. Nests were monitored during the breeding season (May–August) from 2012 through 2014 for at least one full breeding attempt. Some nests were monitored for multiple breeding attempts across >1 years of study (38% of nests). However, it was unknown whether the same breeding pair returned to a given nest between years. Before approaching the nest (~ 1000 m away), we conducted a scan of the nest and the surrounding area for adult Ferruginous Hawks. We assume the hawk’s exceptional vision and the open landscape meant that subjects were alert to our approach at the beginning of each trial. The sex of individuals on the nest was unknown, but males spent very little time on the nest, and the majority of flight initiations were likely by females. We proceeded toward the nest (dependent upon road access and permission from landowners) and documented the horizontal distance (nest height was not incorporated) from the observer to the nest when the individual hawk initiated flight (FID) using handheld GPS and electronic rangefinders. Often, reproductive information could be collected without going directly to the nest, and these distant approaches did not cause flight initiations. Our approach consisted of three possible stages: (1) researchers drove as close to a nest as possible using public roads before (2) parking and exiting the vehicle, and (3) walking the remaining distance to the nest (Fig 1). We were unable to control for approach differences between observers in our study, but nests were approached via the most direct path while maintaining posted speed limits. Thus, inter-observer differences in approach path and speed were assumed to be negligible. The distance from the nest at which these events occurred was calculated in ArcGIS 10.2 [50]. We also calculated the number of previous approaches to the nest. To avoid harming eggs or young, we did not approach nests in cold (< 10°C) or rainy conditions, or if we suspected egg laying had recently occurred. During approaches we exited the home range as soon as possible after necessary data were collected, with the goal of minimizing time spent potentially disturbing the adults. Mean temperature (°C) and wind speed (km/h) were recorded over 60-second intervals using hand held devices (Kestrel; www.kestrelmeters.com) before each approach, at distances > 1 km from the nest. Nest structure was recorded including, trees, man-made Artificial Nesting Platforms (ANPs), transmission tower nests, and few ground nests or buildings (other).

Using ArcGIS 10.2 [50] we mapped all roads (using Alberta Transportation (http://www.transportation.alberta.ca/), Saskatchewan Transportation (http://www.highways.gov.sk.ca/) and IHS Road Layers (2012; www.ihs.com) in a 2500-m radius (approximately the average homerange size; [51]) around each study nest. Our road layer included road classes (highways, range roads and access roads) and surface substrates (hard surface, loose, gravel and dirt), and we used these classes and substrates to create road categories related to vehicle traffic volume. To determine the number of vehicles using these roads categories, we used data from 178 MetroCount 5600 Vehicle Classification Systems (traffic counters) to collect daily traffic volumes (vehicles / day) between the months of May and August from 2008 through 2013, some of which came from a companion Burrowing Owl (*Athene cunicularia*) study [52].

### Statistical analysis

#### Probability of flight initiation from the nest

We used a modified time to event (survival) analysis [53] to model FID, that used the flight initiation from the nest by an adult Ferruginous Hawk as an event of interest. Rather than the conventional analysis interval, time [54], we used investigator distance from nest. The largest observed FID was 950 m and we used 1000 m as our subject entry distance. We assume that we were able to detect Ferruginous Hawks present on the nest with perfect accuracy and assume no left censoring of subjects from the analysis (flight initiation occurred before observation began). However, we did not always approach nests closely enough to cause flight initiation, thus our data were right censored. We binned our flight initiation data into 25-m divisions as a conservative estimate of FID measurement accuracy for GPS, rangefinders and investigators.

We then used the Cox proportional hazards regression model [55], a semiparametric analysis to estimate hazard ratios. Hazard ratios estimate the relative effect of covariates on the hazard function, which is the probability of an event occurring over some interval [53]. In biological terms, we estimated the effect of covariates (hazard ratios) on the relative probability that an adult Ferruginous Hawk (a subject) initiated flight (an event or failure) as we approached the nest. We estimated a shared frailty for each nesting attempt, the Cox regression equivalent of a random intercept model [53], to control for non-independence when repeatedly sampling at the same nesting attempt. The random intercept model served as our null model for human approaches to Ferruginous Hawk nests before fitting covariates. We used the Efron method for handling tied events, flights that occur at identical distances from the nest, to minimizes bias when estimating coefficients for datasets with heavily tied failures [56]. Discrete time-varying covariates, those that changed as we approached the nest, were used to test for an effect of investigator approach type (driving on highways, range roads and access roads, walking and exiting the vehicle) on the probability of flight. We present coefficients generated by the Cox proportional hazard model as hazard ratios (exp[β_i_]) and standard errors. The dependent variable resulting from this analysis was the probability of flight from the nest at a given investigator proximity to the nest, henceforth probability of flight. Probability of flight is analogous to the more commonly measured FID and should be interpreted similarly. For example, a greater estimated probability of flight this is equivalent a higher mean FID estimate from a linear regression model. We also conducted a supplementary analysis using one breeding season for each nest. This analysis was intended to reveal potential biases associated with pseudo-replication when the same adult Ferruginous Hawks returned to nests in multiple years, but we were unable to identify individuals.

#### Vehicle traffic volume quantification

We estimated the daily traffic volume for road class (highway, range road and access roads) and substrate (hard surface, loose, gravel and dirt) combinations using data from traffic counters. Not all class-substrate combinations had significantly different traffic volumes and we combined them to six different types of roads (Table 1). We then extrapolated the estimated traffic volumes for each road type to roads near Ferruginous Hawk nests in our study. This extrapolation is an estimate of traffic volume on each road, as we did not know the actual traffic volumes at each nest. We assigned estimated traffic volume to each road type within 400-m and 2500-m radii around our study nests (henceforth: traffic indices). The 400 m scale was the 95th percentile of our FID data, while the 2500 m scale was based on the average Ferruginous Hawk home range in Canada [51]. Thus, 400 and 2500 m represented the distances at which Ferruginous Hawks would likely respond to, and encounter vehicles in their home range, respectively. Additionally, we predicted that the proximity of passing vehicles was also likely important for Ferruginous Hawk flight initiation, and we applied an inverse weighting to the traffic indices using distance to each road from the nest, such that a weight of 1 was applied to roads adjacent to nests and decreased proportionately to 0 at 400 or 2500 m. The resulting values were an index of traffic volume at each nest at two scales that assigned more importance to vehicular traffic near the Ferruginous Hawk nest (henceforth: near-traffic indices).

**Table 1:** Daily traffic volume (vehicles / day) as recorded by 178 traffic counters installed on roads throughout the range of Ferruginous Hawks in Alberta and Saskatchewan. We present the means ($\bar{\text{x}}$), standard errors (SE), and numbers of roads (n).

Road Type	$\bar{\text{x}}$	SE	n
Hard surface highways	415.2	79.8	22
Loose surface highways	100.2	17.2	8
Hard surface range roads	45.7	20.8	5
Gravel range roads	44.2	4.7	47
Dirt range roads	21.2	3.0	62
Access roads	9.4	2.5	34

#### Model building

We considered covariates for inclusion using forward stepwise model construction with an AIC selection framework [57]. Highly related covariates (r>0.7) were selected for inclusion in our analysis based on the lowest AIC scores when compared. Covariates improving our model fit (ΔAIC>2) were included in sequential model-building steps. Some covariates, such as structure type, wind speed, temperature, year, and ordinal date (Table 2) were potentially important sources of variation in the response variable. Thus, we generated a base model to control for these covariates before testing for effects of stimulus, experience, and landscape context. To construct our stimulus, experience, and landscape models, we used a forward stepwise approach to determine if our variable(s) explained variation in FID, using the base model as a starting point. To test for potential additive and interactive relationships between variables in each model set, we constructed a final model using stimulus, experience and landscape and tested for interactive effects. To assess fit and and to identify high leverage points in our independent variables we used approaches discussed in chapter 11 of [53]. The Nelson-Aalen (NA) cumulative hazard estimator increased linearly with proximity to nest, indicating an increasing likelihood of flight as distance from nest decreased. The Cox-Snell residuals were positively related to the NA estimator (r = 0.99), suggesting good fit of the final model to the data. High leverage points (± 0.02 β), identified by quantifying beta-coefficient changes resulting from the removal of a given data point (DFBETA), were uncommon and their removal did not change covariate significance. All analyses were carried out using Stata v 13 [58].

**Table 2:** Covariates considered for inclusion in a Cox proportional hazards regression model of adult Ferruginous Hawk flight initiation distances when approached by investigators.

Model	Covariate	Abbreviation
Base Model	Nest Structure	struc
	Temperature	temp
	Temperature, quadratic	temp2
	Wind speed	wind
	Wind speed, quadratic	wind2
	Ordinal Date	date
	Ordinal Date, quadratic	date2
	Year	yr
Stimulus	Approach Type^1^	apptype
Experience	Approach Number	appnum
Landscape	Traffic Index 400^†^	ti4
	Traffic Index 2500^†^	ti25
	Near-Traffic Index 400^‡^	nti4
	Near-Traffic Index 2500^‡^	nti25

^1^ a discrete time-varying covariate (see [53]); walking, exiting vehicle, drive highway, drive range road, drive access road

^‡ / †^ Traffic volume on each road within 400 and 2500 m from the nest, each given no weighting (†) and inverse-weighted by distance from nest (‡)

## Results

**Fig 2:**
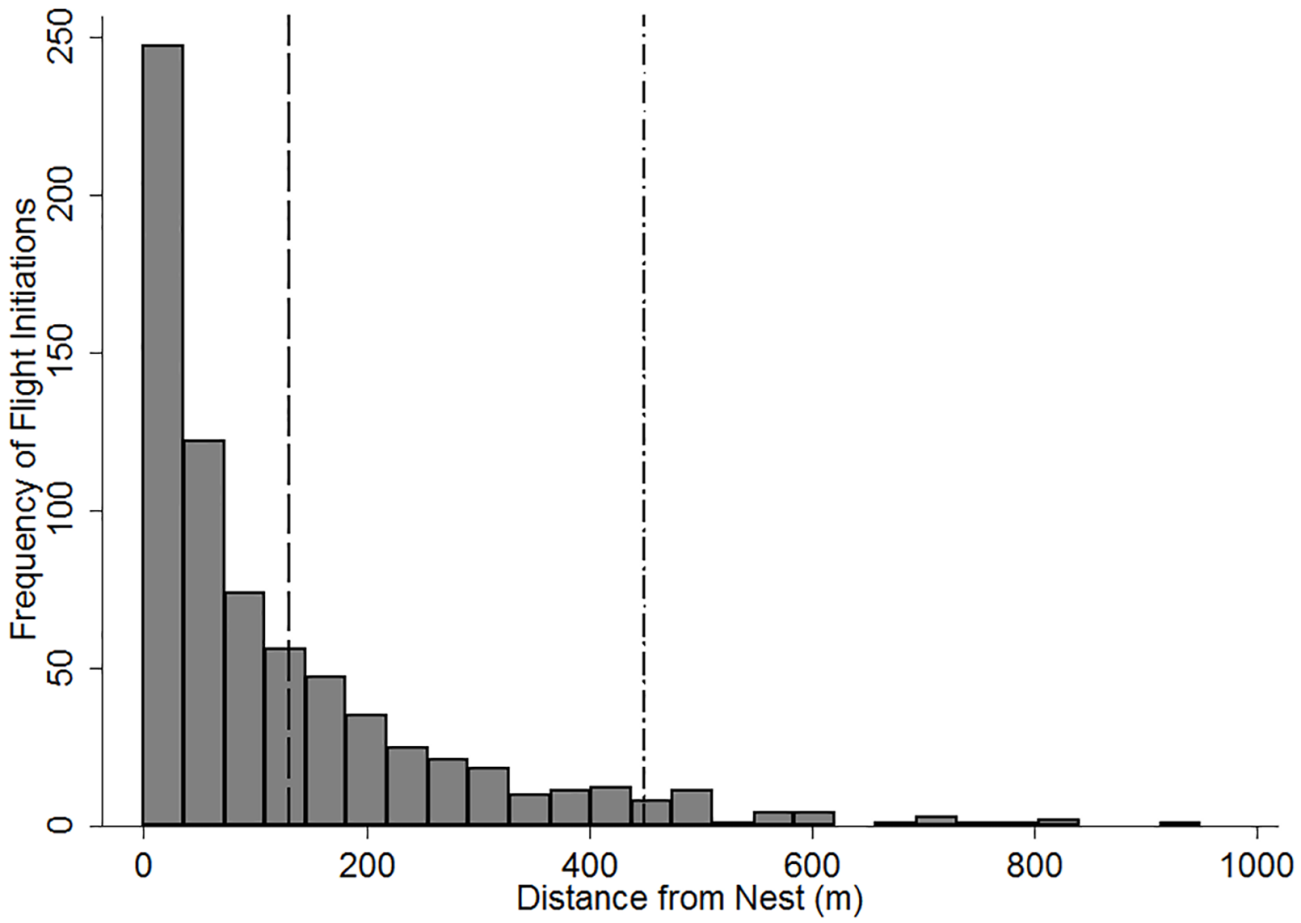
Histogram showing the frequency distribution of Ferruginous Hawk flight initiation distances during investigator nest approaches. Flight initiations were documented in southern Alberta and Saskatchewan from 2012–2014. The dashed and dash-dot vertical lines indicate the mean (130 m) and the 95th percentile (450 m) flight initiation distances, respectively.

At least one adult hawk was present on the nest for 1377 observations, at 623 unique nesting attempts, at 420 different nest sites ($\bar{\text{x}}=1.5\pm 0.03$ SE years sampled per nest). We observed an adult on their nest between one and eight instances for each nesting attempt. We recorded flight initiation by adult Ferruginous Hawks in 721 instances, from 406 unique nesting attempts, at 324 different nests ($\bar{\text{x}}=3.3\pm 0.1$ SE approaches per nesting attempt). Flight initiation distances were highly right-skewed ($\bar{\text{x}}=130\text{ m}$, median = 74 m, 95th percentile = 450 m) and ranged from 0 m to 950 m from the nest (Fig 2).

Linear relationship between flight initiation and both wind speed (ΔAIC = 0.2) and ordinal date (ΔAIC = 1.5) had lower AIC scores than quadratic relationships, and linear terms for these variables were used in subsequent analyses. Flight initiation was related to quadratic temperature (ΔAIC = 5.3), but temperature was highly correlated with linear ordinal date, which received more support (ΔAIC = 47.5). Thus, temperature was not considered in subsequent analyses. Flight initiation was related to nest structure (ΔAIC = 403.2), ordinal date (ΔAIC = 68.5), and year (ΔAIC = 33.6), as identified in forward model building steps 1–3, respectively. Neither wind nor number of young were identified as important for flight initiation in the fourth step (ΔAIC> = 1.1).

Once we created our best baseline, we then added variables to create the best fitting stimulus model, experience model, and landscape model (Table 3). We found evidence that approach type (ΔAIC = 261.1) did improve fit for the stimulus model. Approach number (ΔAIC = 6.4) improved fit for the experience model. The weighted traffic index within 400 m (ΔAIC = 12.3) improved fit for the landscape model. Our full model included approach type (ΔAIC = 261.1), approach number (ΔAIC = 9.4), and the near-traffic (within 400 m) index (ΔAIC = 4.2) in sequential steps. Our power to predict flight initiation was not improved by the inclusion of interactive effects of the learning, stimulus, environment parameters (ΔAIC> = -0.9). as we found no interactive effect of approach type and the near traffic index, Ferruginous Hawks nesting nearer to high traffic volume roads demonstrated equally reduced FID, regardless of the approach type.

**Table 3:** Null, base, stimulus, experience, landscape, and full models created using forward-stepwise model building. We used Cox proportional hazards regression to model the probability of flight by adult Ferruginous Hawks from the nest when approached by an investigator. AIC indicates Akaike's Information Criterion Score [57] used to select for covariates that best fit our data. *h*_*ij*_ (*t*) is the relative hazard at distance from the nest (*d*) given the value of *x* for the *j*^th^ nest approach in the *i*^th^ nesting attempt (the random effect). *α*_*i*_ parameterizes the latent variation between nesting attempts.

Model Name	AIC	Model
Null Model	9345.7	*h*_*ij*_ (*d*) = *h*_*0*_ (*d*) + *α*_*i*_ exp (*x*_*ij*_*β*)
Base Model	8840.4	*h*_*ij*_ (*d*) = *h*_0_ (*d*) + exp *α*_*i*_ (*x*_*ij*_ struc^a^) + exp *α*_*i*_ (*x*_*ij*_ date^b^) + exp *α*_*i*_ (*x*_*ij*_ yr^c^)
Stimulus Model	8569.9	*h*_*ij*_ (*d*) = *h*_0_ (*d*) + exp *α*_*i*_ (*x*_*ij*_ struc^a^) + exp *α*_*i*_ (*x*_*ij*_ date^b^) + exp *α*_*i*_ (*x*_*ij*_ yr^c^) + exp *α*_*i*_ (*x*_*j*_ *β*apptype^d^)
Experience Model	8834.0	*h*_*ij*_ (*d*) = *h*_0_ (*d*) + exp *α*_*i*_ (*x*_*ij*_ struc^a^) + exp *α*_*i*_ (*x*_*ij*_ date^b^) + exp *α*_*i*_ (*x*_*ij*_ yr^c^) + exp *α*_*i*_ (*x*_*j*_ *β*appnum^e^)
Landscape Model	8828.2	*h*_*ij*_ (*d*) = *h*_0_ (*d*) + exp *α*_*i*_ (*x*_*ij*_ struc^a^) + exp *α*_*i*_ (*x*_*ij*_ date^b^) + exp *α*_*i*_ (*x*_*ij*_ yr^c^) + exp *α*_*i*_ (*x*_*j*_ *β*nti4^f^*)*
Final Model	8565.7	*h*_*ij*_ (*d*) = *h*_0_ (*d*) + exp *α*_*i*_ (*x*_*j*_ *β*struc^a^) + exp *α*_*i*_ (*x*_*j*_ *β*date^b^) + exp *α*_*i*_ (*x*_*j*_ *β*yr^c^) + exp *α*_*i*_ (*x*_*j*_ *β*apptype^d^) + exp *α*_*i*_ (*x*_*j*_ *β*appnum^e^) + exp *α*_*i*_ (*x*_*j*_ *β*nti4^f^*)*

^a^ nest structure,

^b^ ordinal date,

^c^ year,

^d^ approach type,

^e^ approach number,

^f^ 400 m near-traffic Index

**Fig 3:**
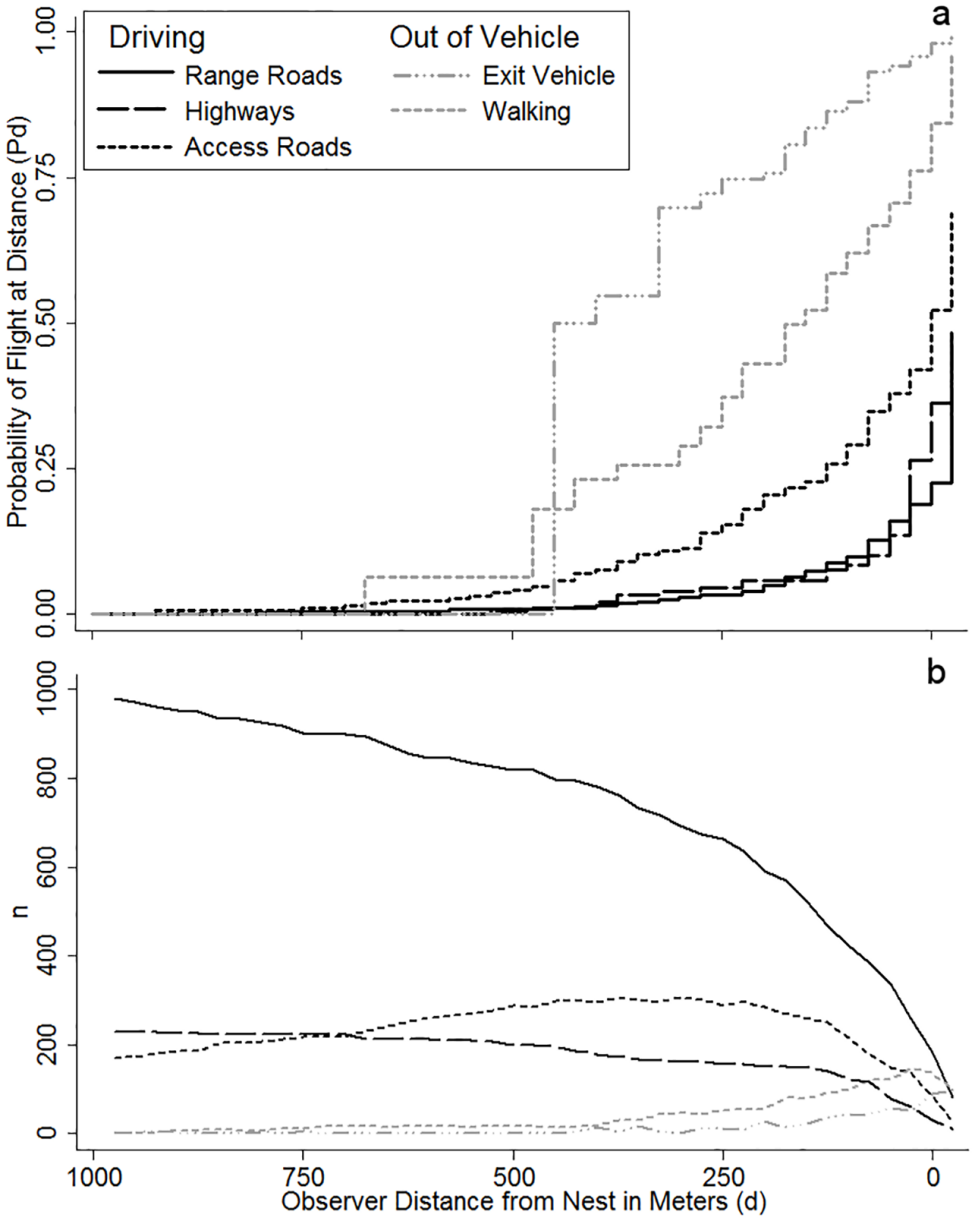
Kaplan-Meier (KM) failure estimator (Kaplan and Meier 1958) used to plot the probability of flight by adult Ferruginous Hawks from the nest at a given distance (a), specifying that they were exposed to investigators at those distances and had not previously initiated flight during the approach, and the sample size (number of instances) for which Ferruginous Hawks were exposed to an approach (b). Separate probability and sample size plots are shown for investigator approaches along highways, range roads and access roads. Additionally, separate plots are shown for walking (approaches on foot) and exiting the vehicle (the act of investigators parking and exiting their vehicle).

**Fig 4:**
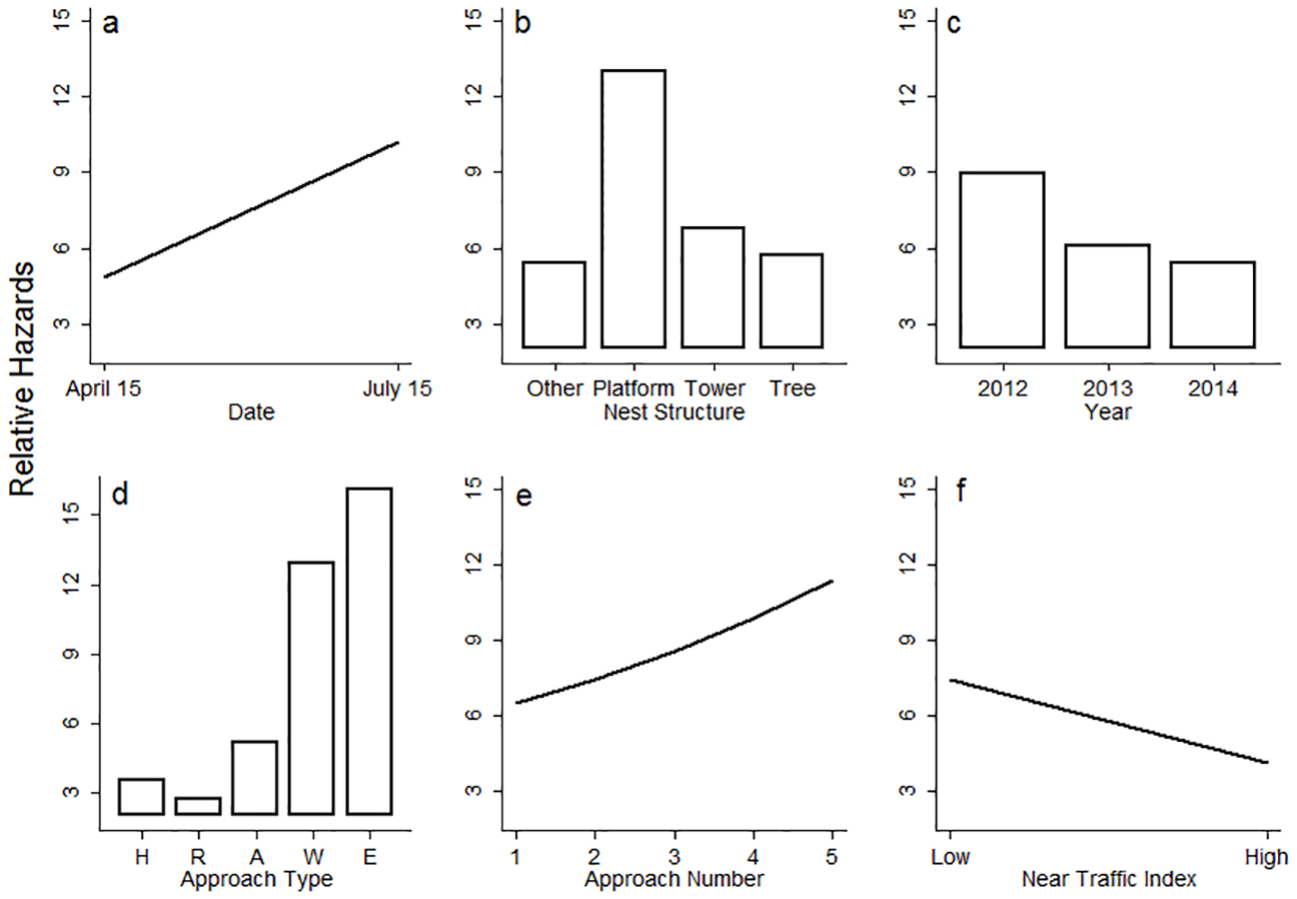
Marginal effects plots demonstrating the effect size, or change to the relative hazards (exp[βi]), defined as relative changes to the probability of flight by adult Ferruginous Hawk from the nest at given distances from the nest, for each covariate in our full model. Each subplot: describes one of six covariates in our full model: a) ordinal date, b) nest structure, c) year, d) approach type, e) approach number, and f) near-traffic index within 4400 m, respectively. H, R, A, W, and E (d) represent driving on highways, range roads, access roads, walking, and exiting the truck, respectively.

Probability of flight initiation by an adult Ferruginous Hawk at a given distance was positively related to ordinal date (1.011 ± 0.003), increasing 1% per day. Probability of flight was more than double (2.7 ± 0.3) for hawks nesting on platforms than for hawks nesting on trees. Probability of flight also differed across years, and was lower in 2013 (0.61 ± 0.06) and 2014 (0.53 ± 0.08) compared to 2012. Approaches by vehicles on range roads resulted in probability of flight about equal to highways (0.70 ± 0.15), but approaches by vehicles on access roads had a probability of flight greater than those on highways (1.3 ± 0.3). Approaches on foot represent a probability of flight nearly four times as great (3.9 ± 0.8) as those on highways, while exiting the vehicle resulted in a probability of flight over five times as great (5.3 ± 1.2) compared to approaches on highways (Fig 3). Walking during approaches were far less common than driving, and we exited the vehicle at most only one distance intervals for a given nest approach, thus these coefficients were estimated based on a smaller number of distances than other approach types. Probability of flight increased by about 18% with each subsequent visit to the nest (1.18 ± 0.06) and decreased as the near-traffic (within 400 m) index increased (0.98 ± 0.01). The largest changes in relative hazards occurred across different approach types and different nest structure types; whereas, the smallest changes were in the near-traffic index and across years (Fig 4). The supplementary analysis using one breeding season for each nest resulted in comparable hazard coefficients and p-values for each covariate (S1 Table). Our shared frailty (random effect; *θ*) for nesting attempt explained a significant proportion of latent variation in our null and base models (*θ* = 0.15 ± 0.06, *P*<0.001).

## Discussion

Mean Ferruginous Hawk FIDs in our study (130 m) were most similar to those in [37] in Colorado, and were shorter on average than all other previous studies, including [38] and [59], where mean FIDs were 393 m and 205 m, respectively. [37] measured FID during the non-breeding season, potentially changing the costs and benefits of flight initiation compared to studies during the breeding season. Additionally, Ferruginous Hawks nested on ground and cliff sites in 96% of nests in [59], and the perception of risk by those individuals may differ from our study. Our data agrees with [37] who found that Ferruginous Hawks approached on foot were more likely to initiate flight than those approached by vehicles and agree with the importance of distinct types of stimuli for explaining variation in FID, as disturbance type had the largest effect size and best relative performance among competing models in our study.

We found, similar to [59], [23], and [24], that Ferruginous Hawks in our study demonstrated significantly reduced probability of flight in response to vehicles compared to humans on foot. Humans on foot, in the form of recreational and agricultural activity are likely infrequent compared to vehicle activity that permeates our study area, with some nests exceeding 5000 vehicles passing/day (http://www.highways.gov.sk.ca/sask-maps). Thus, we suggest that Ferruginous Hawks in our study are more likely to have habituated to vehicles than to humans on foot, and find support for the habituation to vehicles hypothesis. Alternatively, FID in response to humans on foot are more likely to be associated by the disturbed individual with targeted harassment, such as hunting, evoking a stronger behavioural response [16]. Historically, shooting of adults has been an important and common source of mortality for Ferruginous Hawks [40, 60, 61] which may have resulted in an increased aversion to humans on foot, through selection for fearful individuals or behavioural transmission from parents.

Importantly, we found that approaches on roads with lower traffic volumes were more likely to result in flight initiation than those on roads with greater average traffic volumes. Rather than faster moving and noisier approaches on highways and range roads, being perceived as higher risk [20, 21], the likelihood of flight initiation in adult Ferruginous Hawks decreased when approached on highways and range roads; roads that had greater traffic volumes. These results provide additional support for the habituation hypothesis, as vehicles rarely travel on access roads and Ferruginous Hawks are less habituated to vehicle approaches on this road type. We were unable to exclude the possibility that different speeds of travel may have caused differences flight probability between approach types (e.g. Highway speed > range roads > access roads > walking). However, given that our results contradicted previous work that suggested faster and noisier approach speeds are more likely to illicit animal responses [11, 20], we endorse habituation as the more parsimonious mechanism in our study. Approaches that are atypical relative to what animals usually encounter are related to increased behavioural responses. For example, American Robins (*Turdus migratorius*) were more likely to respond to humans walking off paths than those on paths [62], and red kangaroos (*Macropus rufus*) responded with greater FIDs when approached off-trail where humans do not normally walk [23].

The act of exiting the vehicle increased the probability of initiating flight at a given distance more than either humans on foot or driving. This response likely arises from a combination of unmeasured characteristics associated with exiting the vehicle, resulting in an increase in perceived risk. This increasing risk could be related to the time spent in the home range, changing from tangential to direct approach [63], or increasing group size [64] as investigators exit the vehicle. Alternatively, humans likely behave and appear more similar to a predator than does a vehicle, and could be perceived as a predator emerging from hiding. Animals should respond strongly to unfamiliarity, because underestimating risks could result in injury or mortality [4].

Although we propose habituation as the dominant mechanism driving differential Ferruginous Hawk FIDs to stimulus type, we also found evidence for sensitization with repeated researcher approaches. Although avian nest defence tends to increases with nestling age [65, 66], we believe age was statistically accounted for by the ordinal date covariate, and the increase in FID with repeated visitation is a separate effect from nestling age or nest stage. Each successive visit to nests by investigators increased the likelihood that the Ferruginous Hawk would initiate flight from the nest at a given distance. If we interpret greater FIDs as an increased willingness to defend the nest from intruders, this result supports the positive reinforcement hypothesis whereby successfully defending the nest from investigators in one instance resulted in birds initiating flight at a greater distance on subsequent visits, because they had successfully defended the nest in the past [65]. Alternatively, Ferruginous Hawks may have perceived approaches to the nest by investigators as threatening compared to the more common, relatively non-threatening vehicles or humans that did not move directly towards or interact with the nest. Other studies of Ferruginous Hawks showed defending adults increased call rate with repeated visitation to the nest [38]. It is unclear to what extent the Ferruginous Hawks in our study were able to recognize individual vehicles or researchers and subsequently alter their behaviour, but this possibility has been demonstrated in other species [67, 68].

Similar to [38], who showed that Ferruginous Hawks nesting in exurban landscapes demonstrate 43% shorter FIDs than in rural landscapes, we found evidence that individuals nesting in landscapes with greater vehicular traffic near to the nest had a lower probability of flight. The negative relationship between FID and human activity on a landscape has been previously demonstrated in other species [28, 29, 31]. Our study is the first non-urban study to use a quantification of the anthropogenic landscape context around an animal and relate it to flight distance. The decreased probability of flight by Ferruginous Hawks with more traffic near to the nest may reflect habituation to humans around the nest site. Alternatively, individuals with tolerant personalities may choose to build nests where near-traffic index values are greater, and could explain the decreased probability of flight observed in such areas. For example, in other species, individuals with more tolerant personalities nested in regions with more humans [69, 70]. Our study differed from some previous work which has included the starting distance of the approach as a predictor variable [71]. Our exclusion of starting distance is based on the potentially flawed assumption that the animal’s perception that an approach has begun may not correlate with the researcher’s perception. Further, we argue that starting distance is virtually always confounded with a change in transportation type, speed, and/or direction of approach. Thus, we suggest that the method employed in this study, where approaches were assumed to have started from a large distance (1000 m), is a valid alternative.

We suggest date is a proxy for age of young and we found that Ferruginous Hawks were more likely to initiate flight from the nest at a given distance as date increased. Avian nest defence increases across the nesting cycle [65, 66], likely due to increasing value of the young to the parents [65] and increasing offspring survival probabilities with age [72]. The influence of year on probability of flight is likely multipart, but one important source of annual variation may be differential availability of primary prey (Richardson’s Ground Squirrels [*Spermophilus richardsonii*]) across years [73]. For example, scarce prey has been suggested to relate to Ferruginous Hawk sensitivity to human disturbance, potentially owing to changes to their physiological state [39]. The increased probability of flight from ANPs compared to transmission tower or tree nests may be due to the unvegetated, exposed structure of ANPs. Ferruginous Hawks on platform nests may perceive themselves or their young at higher risk of detection by a predator or threat [17], resulting in great probability of flight than concealed nests [74]. Although transmission tower and ANP nests may be similarly exposed, adult hawks may perceive less risk because of the greater height of the transmission tower nests [75]. Nest height presumably decreases nest accessibility by non-avian predators. Although we were unable to quantify height or concealment of nests in our study, future research will benefit from collecting this data, given the potential importance for flight initiation behaviour [76].

Some of the variance in probability of flight was explained by the random intercept in our model, likely owing to unmeasured differences among individuals and nest sites. These differences may include personality [77], body condition [78] stress [79], concealment [17], experience [26, 80], and/or predation risk [81]. Many studies report high inter-individual variation in behavioural responses to humans [65, 68, 82, 83], and delineating sources of inter-individual variation is an area of active study [84–87]. The analysis presented in our study assumed that each breeding attempt was an independent event. Our supplementary analysis generated similar hazard coefficients and p-values (S1 Table) indicating, although breeding attempts are not independent when the same adults return to nests across years, the inclusion of possible return-nesting individuals did not bias our results.

Our study can advise current management strategies used to protect Ferruginous Hawks in Canada. Investigator approaches in our study were both infrequent and short in duration, and likely fall within the low- or medium-disturbance levels for current setback distance recommendations [43]. As only 3% (n = 21) of flight initiations occurred when humans were greater than 500 m from the nest, a 500-m setback for low-impact disturbances is likely sufficient to prevent nearly all flight initiations by Ferruginous Hawks. This applies to most common types of disturbances Ferruginous Hawks likely encounter, such as private or industrial vehicular traffic, recreationists, and private landowners passing by nests. Ultimately, the 1000-m setbacks throughout the breeding season in Alberta are likely overly precautionary for low- or medium-disturbance levels.

Our study highlighted the complex and dynamic interactions between costs and benefits comprising a decision to react to an approaching human. FID has been quantified previously in Ferruginous Hawks [38], other raptors [37], and other species [16]. FID has been used by government regulators to establish setback distances [2, 88, 89], which are used to limit industrial activity near Ferruginous Hawks nests in Canada [43] to minimize human-wildlife conflicts. A powerful approach for future studies would be the use of FID or probability of flight in conjunction with fitness or population level information to understand how animal decision making may influence long term viability of the population. In agreement with [15], we suggest that, when attempting to reduce the incidence of wildlife flight initiation, consideration should be given to the stimulus, the individual, and the environmental (in this case anthropogenic) context.

## Supporting information

S1 Table Relative hazard coefficients (± standard errors) and p-values from the Cox proportional hazard survival model of all Ferruginous Hawk flight initiations collected from 2012 to 2014 (Full Dataset) and a subset of data that includes only one year of study for each nest (Subset). The Full Dataset includes 1377 nest approaches across 623 breeding attempts at 420 different nests. The data Subset includes 985 nest approaches across 420 breeding attempts at 420 different nests.
(DOCX)
