# Supplementary material for: Flight initiation by Ferruginous Hawks depends on disturbance type, experience, and the anthropogenic landscape
Source: PLoS One. 2017 May 18;12(5):e0177584. doi: 10.1371/journal.pone.0177584 (PMC5436750; doi:10.1371/journal.pone.0177584)
Supplement: S1 Table — The Full Dataset includes 1377 nest approaches across 623 breeding attempts at 420 different nests. The data Subset includes 985 nest approaches across 420 breeding attempts at 420 different nests. (DOCX) [file pone.0177584.s001.docx]

S1 Table: Relative hazard coefficients (± standard errors) and p-values from the Cox proportional hazard survival model of all Ferruginous Hawk flight initiations collected from 2012 to 2014 (Full Dataset) and a subset of data that includes only one year of study for each nest (Subset). The Full Dataset includes 1377 nest approaches across 623 breeding attempts at 420 different nests. The data Subset includes 985 nest approaches across 420 breeding attempts at 420 different nests.

|  |  | Full Dataset | | Subset | |
| --- | --- | --- | --- | --- | --- |
|  |  | Relative Hazard ± SE | *p* | Relative Hazard ± SE | *p* |
| Covariate |  |  |  |  |  |
|  |  |  |  |  |  |
| Nest Structure | Tree* |  |  |  |  |
|  | Other | 0.87 ± 0.21 | 0.56 | 0.86 ± 0.25 | 0.59 |
|  | Platform | 2.75 ± 0.33 | <0.001 | 3.11 ± 0.46 | <0.001 |
|  | Transmission tower | 1.17 ± 0.29 | 0.54 | 1.17 ± 0.36 | 0.6 |
|  |  |  |  |  |  |
| Ordinal Date |  | 1.01 ± 0.003 | <0.001 | 1.00 ± 0.003 | 0.06 |
|  |  |  |  |  |  |
|  |  |  |  |  |  |
| Year | 2012* |  |  |  |  |
|  | 2013 | 0.61 ± 0.06 | <0.001 | 0.57 ± 0.07 | <0.001 |
|  | 2014 | 0.53 ± 0.81 | <0.001 | 0.53 ± 0.12 | 0.01 |
|  |  |  |  |  |  |
|  |  |  |  |  |  |
| Approach Type | Highway* |  |  |  |  |
|  | Range Road | 0.7 ± 0.15 | 0.1 | 0.72 ± 0.18 | 0.19 |
|  | Access Road | 1.33 ± 0.29 | 0.19 | 1.33 ± 0.33 | 0.25 |
|  | Walk | 3.91 ± 0.84 | <0.001 | 4.1 ± 1.01 | <0.001 |
|  | Exit Truck | 5.30 ± 1.16 | <0.001 | 5.29 ± 1.32 | <0.001 |
|  |  |  |  |  |  |
| Approach Number |  | 1.18 ± 0.06 | <0.001 | 1.24 ± 0.07 | <0.001 |
|  |  |  |  |  |  |
| Near-traffic index |  | 0.98 ± 0.01 | 0.02 | 0.98 ± 0.01 | 0.03 |
|  |  |  |  |  |  |
| Random Intercept Breeding Attempt (θ) |  | 0.15 ± 0.06 |  | 0.19 ± 0.07 |  |
| * Indicates the baseline categorical variable | | |  |  |  |
